# The causal relationship between 91 inflammatory cytokines and chronic pancreatitis, and the mediating role of 1400 metabolites

**DOI:** 10.1097/MD.0000000000043880

**Published:** 2025-09-19

**Authors:** Chengning Yang, Yuqing Wang, Lijian Liu, Zuomei Luo, Nan Chen, Zhu Liu, Liqun Li, Guangwen Chen

**Affiliations:** a First Affiliated Hospital of Guangxi University of Chinese Medicine, Nanning City, Guangxi Province, China; b Graduate School of Guangxi University of Chinese Medicine, Nanning City, Guangxi Province, China.

**Keywords:** bidirectional 2 sample Mendelian randomization, chronic pancreatitis, inflammatory cytokines, metabolites

## Abstract

Considerable amounts of studies have confirmed a close relationship between specific inflammatory cytokines and chronic pancreatitis (CP), while the causal effect between the 2 remains unclear. This study is to evaluate the causal relationship between 91 inflammatory cytokines and CP using bidirectional 2-sample Mendelian randomization (MR) method, and to investigate the mediating role of 1400 metabolites through a 2-step MR analysis. Genome wide association study (GWAS) data related to 91 inflammatory cytokines were sourced from 14,824 participants of European populations, and CP related GWAS data from a Finnish database, covering 3875 cases of CP cases and 361,641 controls. A total of 1400 circulating metabolites were derived from 8299 individuals. This study used inverse variance weighted (IVW) as the main analysis method, complemented by 4 other methods. In addition, sensitivity analysis was conducted at different levels, including Cochran *Q* statistics, MR-egger intercept, MR-PRESSO global test, and “leave-one-out method” (LOO) analysis, ensuring the robustness of the results. The IVW method revealed that levels of CCL23 [OR = 1.120, 95% CI: 1.014–1.237, *P* = .026], DNER [OR = 1.151, 95% CI: 1.020–1.300, *P* = .023], IL-6 [OR = 1.240, 95% CI: 1.034–1.486, *P* = .020], and TNFRSF9 [OR = 1.156, 95% CI: 1.020–1.309, *P* = .023] increased the CP risk; while CCL19 [OR = 0.902, 95% CI: 0.820−0.991, *P* = .033], IFN-gamma (IFN-γ) [OR = 0.847, 95% CI: 0.734−0.977, *P* = .023], IL-10 [OR = 0.853, 95% CI: 0.736−0.988, *P* = .034], IL-2 [OR = 0.836, 95% CI: 0.719−0.972, *P* = .020], MCP-3 [OR = 0.879, 95% CI: 0.779−0.993, *P* = .038] had a protective effect on CP. Although mediation analysis identified 13 metabolites mediating the causal relationships between CCL19, CCL23, IFN-gamma, IL-2, IL-6, MCP-3, and CP, none of these mediating effects achieved statistical significance (*P* > .05). The 2-sample MR analysis in this study provided convincing evidence for the causal effects of circulating inflammatory cytokines on CP, confirming that CCL23, DNER, IL-6, and TNFRSF9 can increase the CP risk, while CCL19, IFN-gamma, IL-10, IL-2, and MCP-3 reduce the CP risk. The evidence was insufficient to prove a direct mediating role of metabolites in the causal relationship between inflammatory cytokines and CP. This study may contribute to a better understanding of the pathogenesis of CP and improve its prevention and treatment.

## 1. Introduction

Chronic pancreatitis (CP) is a progressive inflammatory disease characterized by pathological fibrosis of the pancreas.^[1]^ The global epidemiological survey in 2020 showed a incidence rate of CP about 9.62 cases per 100,000 people, and a prevalence rate ranging from 13.5 to 560 cases per 100,000 people.^[2]^ The statistics in 2022 revealed that the annual incidence rate of CP in the United States ranges from 5 to 8 cases per 100,000 people, and a prevalence rate of 42 to 73 cases per 100,000 people.^[3]^ CP manifests not as a single inflammatory disease but a complex syndrome characterized by chronic abdominal pain, fatty diarrhea, impaired glucose tolerance, weight loss, and malnutrition,^[2]^ with 80% to 90% of patients experiencing pain as the main symptom.^[4]^ There are challenges in clinic diagnosis and treatment of CP, with treatments focusing primarily on symptomatic relief such as pain management and antidiarrheal measures, rather than reversing disease progression. Although the opioid drugs can effectively alleviate pain, the long-term use may lead to serious consequences, including the anesthesia-induced intestinal syndrome and opioid-induced hyperalgesia.^[5]^ Even proved to be a benign disease, CP has a long progress and stubborn symptoms, often accompanied by complications such as diabetes, pancreatic exocrine insufficiency, and metabolic bone disease,^[6]^ posing further risks to health. According to research reports, the 10-year mortality rate of patients with CP ranges from 13.7% to 34%, higher than that of common cancers.^[7]^ Therefore, further exploration to the etiology and pathogenesis of CP becomes necessary to provide effective prevention and health management strategies to scientifically assist in the treatment of this disease as a global diagnostic and therapeutic challenge.

The etiology and pathogenesis of CP have not been fully revealed, while research has confirmed the interaction between CP and inflammatory cytokines. Studies have shown that inflammatory cytokines such as tumor necrosis factor-α (TNF-α), interleukin-6 (IL-6), interleukin-1 (IL-1), monocyte chemotactic protein-1 (MCP-1), macrophage inflammation protein-1α (MIP-1α), leukemia infection factor, amphiregulin protein, plateau derived growth factor-α (PDGF-α), and transforming growth factor-β1 (TGF-β1) can stimulate the proliferation and differentiation of pancreatic stellate cells into myofibroblasts, exacerbate pancreatic fibrosis, and play a crucial role in the pathological process of CP.^[8–11]^ In addition, IL-10 has been found to inhibit pancreatic fibrosis, thereby protecting CP.^[12]^ However, these causal relationships have been established through clinical observational studies, with many unpredictable potential confounding factors. Therefore, these causal relationships may be influenced by the reverse causal effect and confounding factors, leading to biased results. Therefore, a reliable and robust inference method is needed to clarify the causal relationship among the 3.

Mendelian randomization (MR) is a novel epidemiological method using genetic variants as the instrumental variable to reveal causal associations between the exposure and the outcome.^[13]^ The fundamental concept of MR is to infer the impact of gene phenotypes on diseases by the randomly allocated genes in natural environments. While controlling for potential confounding factors and avoiding reverse causal bias, MR, acting as a “randomizer,”^[14]^ randomly assigns the effects of the exposure to individuals. By analyzing the relationship between genetic variation and the outcome, the causal effect of the exposure on the outcome could be concluded. Based on previous research, this study proposed the scientific hypothesis: Is there a causal relationship between circulating inflammatory cytokines and CP? To our knowledge, bidirectional 2-sample MR has not yielded high-level evidence to answer the question. Research has reported that toxic metabolite stress can lead to the onset of CP.^[15]^ Three metabolite biomarkers of arginine, lysophosphatidylcholine, and high sensitivity C reactive protein have potential roles in systemic inflammation.^[16]^ However, the mediating role of circulating metabolites remains unknown. Therefore, this study revealed the causal relationship between inflammatory cytokines and CP with bidirectional 2-sample MR, and evaluated the mediating effect of circulating metabolites in the causal relationship between the 2 through 2-step MR, providing scientific insight into the diagnosis, treatment and prevention of CP.

## 2. Materials and methods

### 2.1. Study design

**Figure 1.**
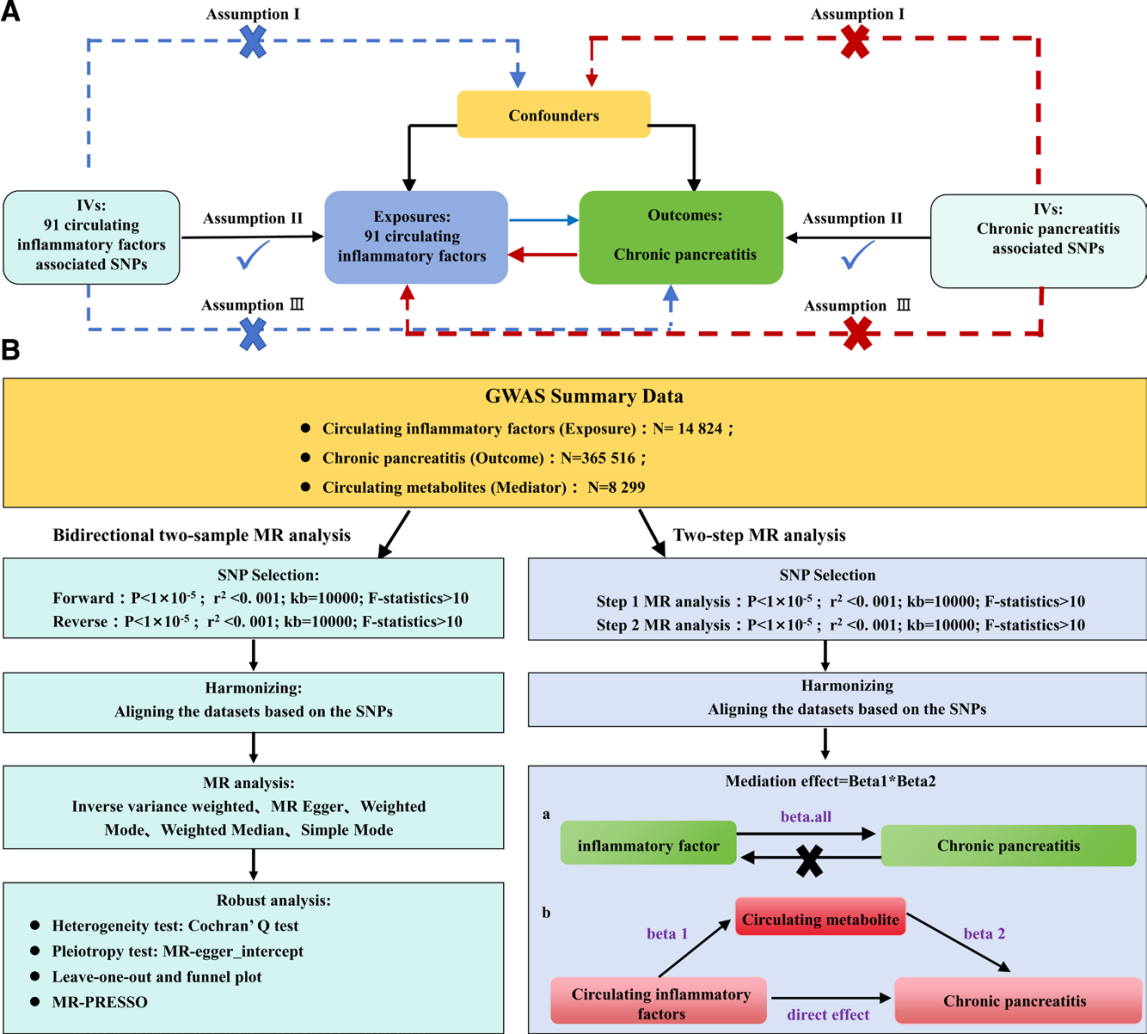
Assumptions and study design of the bidirectional MR study of 91 circulating inflammatory factors in association with CP. CP = chronic pancreatitis, MR = Mendelian randomization, SNPs = single nucleotide polymorphisms.

The study must adhere to 3 core assumptions: Independence: The selected single nucleotide polymorphisms (SNPs) are independent of any confounding factors. Correlation: The selected SNPs are significantly correlated with the exposure. Exclusivity: SNPs merely affect the outcome through the exposure. We investigated the causal relationship between CP and the circulating inflammatory cytokines by sequentially selecting SNPs associated with the 2. Finally, a 2-step MR analysis was applied to infer the mediating role of 1400 blood metabolites in their causal relationship. Our data were sourced from the summary statistics of public database genome wide association study (GWAS). These data have already obtained relevant ethical approval in the original study; thus, no additional ethical application is needed. Meanwhile, the research followed the MR report standard of strengthening the reporting of observational studies in epidemiology–Mendelian randomization (STROBE-MR).^[17]^ The study design is shown in Figure 1.

### 2.2. Data sources

GWAS data for 91 circulating inflammatory cytokines were downloaded from https://www.phpc.cam.ac.uk/ceu/proteins (ID: GCST90274758 to GCST90274848). This study consisted of 11 cohorts, including 14,824 European individuals, and detailed information could be found in the original paper.^[18]^ GWAS summary data for CP were downloaded from FinnGen (https://www.finngen.fi/en)^[19]^ (ID: finngen.R10_K11_CRONPANC.gz). This study covered 365,516 participants, including 3875 CP cases and 361,641 controls. GWAS data for 1400 blood metabolites were accessed from NHGRI-EBI GWAS Catalog (https://www.ebi.ac.uk/gwas/; ID: CST90199621–902010209). This study involved 8299 individuals of European ancestry^[20]^ and there is no sample overlap, avoiding bias by racial differences.

### 2.3. Genetic IVs selection

To ensure (1) the exposure are strongly correlated to the outcome variants; (2) the exposure is highly independent to any possible confounding factors, we initially selected SNPs with significance as IVs, using *P* < 1e−6 and *P* < 1e−7 as screening condition. However, the screened SNPs were less than the minimum quantity to perform convincible MR analysis. Therefore, we adjusted the screening threshold of SNPs for inflammatory cytokines, CP, and metabolites to *P* < 1e−5. Parameters for removing linkage disequilibrium were set as *r*^2^ = 0.001 and clump = 10,000 kb to ensure the independence of SNPs and avoid bias. Parameter for screening out weak IVs was set as *F* > 10 to ensure the SNPs strength. Detailed characteristics of SNPs are shown in Tables S1 to S3 (Supplemental Digital Content, https://links.lww.com/MD/P695).

### 2.4. Bidirectional MR analysis

A bidirectional 2-sample MR analysis was conducted to evaluate the causal relationship between 91 inflammatory cytokines and CP, employing 5 statistical methods of inverse variance weighted (IVW), MR-egger, Weighted Mode, Weighted Medium, and Simple Mode. The IVW method was used to determine the results and evaluate the significance of causal relationship when the results from these 5 methods were inconsistent.^[13]^ In addition, sensitivity analysis was conducted using different methods. Cochran *Q* test was adopted to determine the heterogeneity of SNPs, and *P* < .05 indicating significant heterogeneity. In that case, MR analysis was performed with random-effect model. In addition, to ensure the independence assumption, MR-Egger intercept was applied to evaluate the horizontal pleiotropy of SNPs, confirming the independence of SNPs from confounding factors.^[21]^ A *P* < .05 indicated the existence of horizontal pleiotropy. If so, we employed Mendelian randomization pleiotropy residual sum and outlier (MR-PRESSO) to detect outliers, and reanalyzed after removing outliers to further detect the horizontal pleiotropy.^[22]^ “Leave-one-out” sensitivity analysis was used for evaluating the impact of individual SNP on the overall causal relationship. Data analysis was conducted with the “TwoSampleMR” package from R software (version R 4.3.3).

### 2.5. Mediation analysis

Two-step MR was utilized for further mediation analysis to investigate the mediating effects of 1400 metabolites in the causal relationship between inflammatory cytokines and CP. Firstly, MR analysis was conducted based on SNPs of inflammatory cytokines and metabolites to evaluate the impact of inflammatory cytokines on metabolite levels (beta1). Then, MR analysis was performed on the SNPs of metabolites and CP to investigate the impact of metabolite levels on CP risk (beta2). Finally, based on the bidirectional 2-sample MR results, eligible inflammatory cytokines (inflammatory cytokines have significant impacts on CP without reverse causation or pleiotropy) were selected to evaluate the mediating effect [formula: the mediating effect (Beta1*2) = Beta 1* Beta2].

## 3. Results

### 3.1. Bidirectional 2 sample MR analysis

#### 3.1.1. Causal effect of circulating inflammatory cytokines on CP

**Figure 2.**
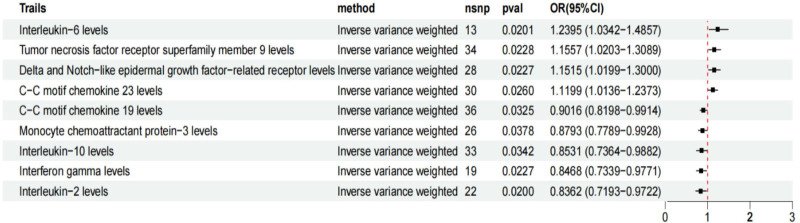
Forest plots of the causal association between CCL23, DNER, IL-6, TNFRSF9, CCL19, IFN-gamma, IL-10, IL-2, and MCP-3 and CP in the result in the forward MR analysis. CCL23 = C-C motif chemokine 23, CCL19 = C-C motif chemokine 19, DNER = delta and notch-like epidermal growth factor-related receptor, IFN-gamma = interferon gamma, IL-2 = interleukin-2, IL-6 = interleukin-6, IL-10 = interleukin-10, MCP-3 = monocyte chemoattractant protein-3, NSNP = the number of single nucleotide polymorphism, TNFRSF9 = tumor necrosis factor receptor superfamily member 9.

**Figure 3.**
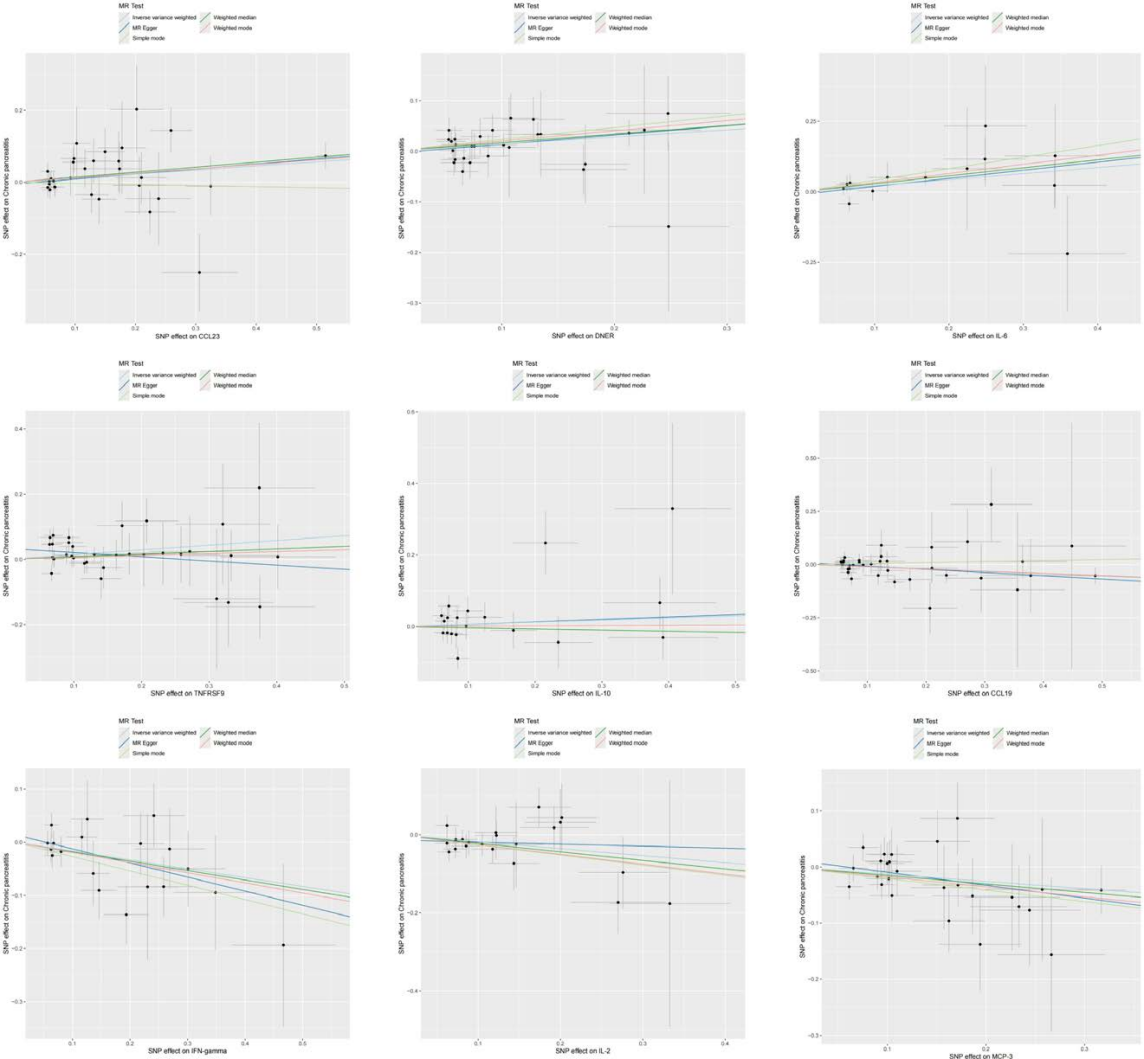
Scatter plot of the positive causal relationship between CCL23, DNER, IL-6, TNFRSF9, IL-10, CCL19, IFN-gamma, IL-2, and MCP-3 and CP. CCL23 = C-C motif chemokine 23, CCL19 = C-C motif chemokine 19, DNER = delta and notch-like epidermal growth factor-related receptor, IFN-gamma = interferon gamma, IL-2 = interleukin-2, IL-6 = interleukin-6, IL-10 = interleukin-10, MCP-3 = monocyte chemoattractant protein-3, TNFRSF9 = tumor necrosis factor receptor superfamily member 9.

The aforementioned MR analysis was performed to evaluate the impact of inflammatory cytokines on CP, with different results listed in Table S4 (Supplemental Digital Content, https://links.lww.com/MD/P695). Specifically, the preliminary results of IVW revealed a positive causal effect of 4 inflammatory cytokines levels on CP, including: C-C motif chemotherapy 23 (CCL23) [OR = 1.120, 95% CI: 1.014–1.237, *P* = .026], delta and notch-like inflammatory growth factor-related receptor (DNER) [OR = 1.151, 95% CI: 1.020–1.300, *P* = .023], interleukin-6 (IL-6) [OR = 1.240, 95% CI: 1.034–1.486, *P* = .020] [OR = 1.156, 95% CI: 1.020–1.309, *P* = .023]. Besides, we also found a negative causal association between 5 inflammatory cytokines levels and CP, including: C-C motif chemotherapy 19 (CCL19) [OR = 0.902, 95% CI: 0.820–0.991, *P* = .033], interferon gamma (IFN-gamma) [OR = 0.847, 95% CI: 0.734–0.977, *P* = .023], interleukin-10 (IL-10) [OR = 0.853, 95% CI: 0.736–0.988, *P* = .034], interleukin-2 (IL-2) [OR = 0.836, 95% CI: 0.719–0.972, *P* = .020], monocyte chemoattractant protein-3 (MCP-3) [OR = 0.879, 95% CI: 0.779–0.993, *P* = .038] (Fig. 2). The scatter plot displayed that CCL23, DNER, and IL-6 factors increased the CP risk, while CCL19, IFN-gamma, IL-10, IL-2, and MCP-3 factors reduced the CP risk, and TNFRSF9 had a bidirectional effect on CP (Fig. 3). Except for IL-10, the Cochran *Q* test for the other 8 causal relationships did not show heterogeneity (*P* > .05) (Table S8, Supplemental Digital Content, https://links.lww.com/MD/P695). Despite slight heterogeneity, IL-10 exhibited consistent results under a random-effect model, remaining statistically significant. None of the other inflammatory cytokines except TNFRSF9 showed evidence of pleiotropy based on the MR-egger intercept and MR-PRESSO test (*P* > .05). Although the MR-egger intercept results suggested potential horizontal pleiotropy for TNFRSF9 (*P* = .022), this was resolved by excluding outliers with MR-PRESSO (*P* = .156). Overall, the sensitivity analysis results confirmed the robustness of these causal relationships.

#### 3.1.2. The effect of CP on inflammatory cytokines

**Figure 4.**
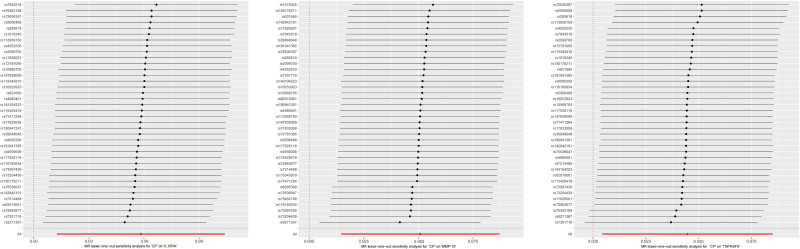
“Leave-one-out method” of the reverse causal relationship between CP and IL15RA, MMP-10, and TNFRSF9. IL15RA = interleukin-15 receptor subunit alpha, MMP-10 = matrix metalloproteinase-10, TNFRSF9 = tumor necrosis factor receptor superfamily member 9.

**Figure 5.**
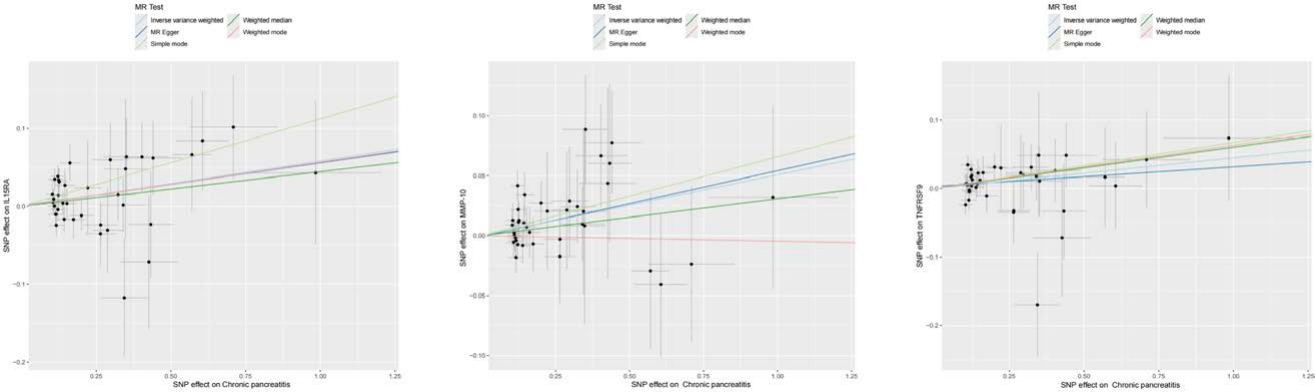
Scatter plot of the reverse causal relationship between CP and IL15RA, MMP-10, and TNFRSF9. CP = chronic pancreatitis, IL15RA = interleukin-15 receptor subunit alpha, MMP-10 = matrix metalloproteinase-10, TNFRSF9 = Tumor necrosis factor receptor superfamily member 9.

In reverse MR analysis, all selected SNPs were robust IVs. The IVW results showed that CP increased the levels of 3 inflammatory cytokines, including interleukin-15 receptor subunit alpha (IL15RA) [OR = 1.060, 95% CI: 1.013–1.110, *P* = .012], matrix metalloproteinase-10 (MMP-10) [OR = 1.052, 95% CI: 1.015–1.091, *P* = .006], TNFRSF9 [OR = 1.046, 95% CI: 1.004–1.089, *P* = .030], which were positively correlated with CP (Fig. 4). The scatter plot displayed that CP increased the levels of IL15RA, MMP-10, and TNFRSF9 (Fig. 5), with no heterogeneity observed in the Cochran *Q* test (Table S9, Supplemental Digital Content, https://links.lww.com/MD/P695), and no evidence of horizontal pleiotropy (*P* > .05) in the MR-egger intercept and MR-PRESSO methods (Table S5, Supplemental Digital Content, https://links.lww.com/MD/P695).

### 3.2. Two-step MR analysis

#### 3.2.1. Effects of inflammatory cytokines on metabolites

All selected SNPs regarding metabolites were robust IVs, and the causal effects of 6 inflammatory cytokines on 13 metabolites were identified using IVW. As shown in Table S6 (Supplemental Digital Content, https://links.lww.com/MD/P695) positive correlations between CCL19 and X-16964 (*P* = .048), Adenosine 5’-monophosphate (AMP) to isoleucine ratio (*P* = .030), AMP to urate ratio (*P* = .044) were statistically significant. Similarly, there were positive correlations between CCL23 and 1-arachidonoyl-gpc (20:4n6) (*P* = .018), X-12026 (*P* = .027), X-23639 (*P* = .019), X-24947 (*P* = .038), as well as between the levels of IL-2 and anthranilate (*P* = .049).

We also found negative correlations between CCL23 and the uridine to pseudouridine ratio (*P* = .042), the levels of IFN-gamma and 1-arachidonoyl-gpc (20:4n6) (*P* = .037) as well as X-16964 (*P* = .030), IL-6 and X-23639 (*P* = .036), the levels of MCP-3 and arachidonate (20:4n6) (*P* = .021). Except for CCL19 and AMP to urate ratio (*P* = .094), no significant heterogeneity was observed in the Cochran *Q* test for other causal relationships. No statistical significance found in MR-Egger intercept and MR-PRESSO tests indicated no horizontal pleiotropy (Table S6, Supplemental Digital Content, https://links.lww.com/MD/P695).

#### 3.2.2. Effects of metabolites on CP

After the analysis of 1400 metabolites, 69 of them showed significant causality with CP, exhibiting no heterogeneity or horizontal pleiotropy (Table S7, Supplemental Digital Content, https://links.lww.com/MD/P695). Among the 69 robust causal relationships, 38 metabolites, including 4-methyl-2-oxopentanoate (*P* = .049), isovalerate (i5:0) (*P* = .035), 1-methylhistidine (*P* = .002), alpha-hydroxyisovalerate (*P* = .020), 1-linolyl-GPE (18:2) (*P* = .021) exhibited positive correlations with CP, while the other 31 metabolites, including 4-hydroxyhippurate (*P* = .009), 1-arachidonoyl-gpc (20:4n6) (*P* = .009), N-acetylserine (*P* = .046), Sphinganine-1-phase (*P* = .008), Ergothioneine (*P* = .040), were negatively associated with CP. The Cochran *Q* test showed no heterogeneity in the other 67 causal relationships, except for N-acetyl-L-glutamine and N-delta-acetylornithine. No pleiotropy was observed in the MR-egger intercept tests for other inflammatory cytokines (*P* > .05), while 4-hydroxyhippurate was detected presenting horizontal pleiotropy (*P* = .022), which disappeared after removing outliers through MR-PRESSO (*P* = .202). The MR-PRESSO results indicated horizontal pleiotropy in N-acetyl-L-glutamine (*P* = .047).

#### 3.2.3. Mediating effect

When conducting mediation analysis on 1400 metabolites, 6 metabolites were identified as mediators in relationship between inflammatory cytokines and CP. Inflammatory cytokines and corresponding metabolites are as follows: CCL19 (X-16964, AMP to isoleucine ratio, AMP to urate ratio), CCL23 (1-arachidonoyl-gpc (20:4n6), X-12026, X-23639, X-24947, uridine to pseudouridine ratio), IFN-gamma (1-arachidonoyl-gpc (20:4n6), X-16964), IL-2 (anthranilate), IL-6 (X-23639), MCP-3 (arachidonate (20:4n6)). Although these metabolites were found influenced by inflammatory cytokines and to affect CP, their unsignificant mediating effect (*P* > .05) indicated that the aforementioned metabolites may not mediate these causal relationships (Table 1).

**Table 1:** Mediating effect.

Inflammatory cytokines	Mediator	The effect of exposure on outcome, β (95% CI)	The effect of exposure on mediator, β1 (95% CI)	The effect of mediator on outcome, β2 (95% CI)	Mediating effect	*P*
C-C motif chemokine 19 levels	X-16964 levels	0.902 (0.820–0.991)	1.087 (1.001–1.180)	0.811 (0.702–0.936)	−0.0175 (−0.0385 to 0.00358)	.104
	Adenosine 5’−monophosphate (AMP) to isoleucine ratio	0.902 (0.820–0.991)	1.096 (1.009–1.191)	0.906 (0.842–0.974)	−0.0091 (−0.0197 to 0.00148)	.092
	Adenosine 5’−monophosphate (AMP) to urate ratio	0.902 (0.820–0.991)	1.097 (1.003–1.199)	1.178 (1.052–1.320)	0.0151 (−0.00294 to 0.0332)	.101
C-C motif chemokine 23 levels	1-arachidonoyl-gpc (20:4n6) levels	1.120 (1.014–1.237)	1.079 (1.013–1.148)	0.895 (0.823–0.973)	−0.00843 (−0.0179 to 0.00101)	.080
	X-12026 levels	1.120 (1.014–1.237)	1.078 (1.009–1.153)	0.845 (0.750–0.951)	−0.0127 (−0.0271 to 0.00167)	.083
	X-23639 levels	1.120 (1.014–1.237)	1.079 (1.012–1.149)	1.186 (1.068–1.316)	0.0129 (−0.000472 to 0.0263)	.059
	X-24947 levels	1.120 (1.014–1.237)	1.069 (1.004–1.139)	1.092 (1.026–1.163)	0.00588 (−0.00108 to 0.0128)	.098
	Uridine to pseudouridine ratio	1.120 (1.014–1.237)	0.940 (0.885–0.998)	0.799 (0.708–0.901)	0.014 (−0.00145 to 0.0294)	.076
Interferon gamma levels	1-arachidonoyl-gpc (20:4n6) levels	0.847 (0.734–0.977)	0.894 (0.804–0.993)	0.895 (0.823–0.973)	0.0125 (−0.00257 to 0.0276)	.104
	X-16964 levels	0.847 (0.734–0.977)	0.887 (0.796–0.988)	0.811 (0.702–0.936)	0.0251 (−0.00334 to 0.0536)	.084
Interleukin-2 levels	Anthranilate levels	0.836 (0.719–0.972)	1.158 (1.001–1.339)	0.891 (0.824–0.963)	−0.0169 (−0.0373 to 0.00343)	.103
Interleukin-6 levels	X-23639 levels	1.240 (1.034–1.486)	0.881 (0.783–0.992)	1.186 (1.068–1.316)	−0.0216 (−0.0457 to 0.00256)	.080
Monocyte chemoattractant protein-3 levels	Arachidonate (20:4n6) levels	0.879 (0.779–0.993)	0.909 (0.838–0.985)	0.863 (0.782–0.952)	0.0141 (−0.00112 to 0.0293)	.069

CCL19 = C-C motif chemokine 19, CCL23 = C-C motif chemokine 23, IFN-gamma = interferon gamma, IL-2 = interleukin-2, IL-6 = interleukin-6, MCP-3 = monocyte chemoattractant protein-3.

## 4. Discussion

This study, involving the largest collection of GWAS data on circulating inflammatory cytokines and metabolites so far, conducted a bidirectional 2-sample and 2-step MR survey to evaluate the causal relationship between 91 inflammatory cytokines and CP, and to analyze the potential mediating role of 1400 metabolites. The analysis results showed that CP risk, positively correlated with CCL23, DNER, IL-6, TNFRSF9, while negatively associated with CCL19, IFN-gamma, IL-10, IL-2, MCP-3, and increased TNFRSF9 levels. The mediation analysis showed that although several metabolites were found influenced by inflammatory cytokines and to affect CP, their mediating effects were not significant (*P* > .05). Sensitivity analysis further strengthened the robustness of the results, providing a scientific reference for future prevention and treatment strategies for CP.

C-C chemokine ligand 23 (CCL23), also known as myeloid progenitor inhibitory factor 1 or macrophage inflammatory protein, acts as a pivotal chemokine involved in inflammation and host defense response.^[23]^ It recruits and attracts immune cells to injury or infection sites, stimulates the release of pro-inflammatory cytokines, and induces the expression of adhesion molecules, thereby participating in the inflammatory response.^[24]^ CCL23 serves as a biomarker involved in the progression of various inflammatory diseases. A key pathogenesis of CP involves the release of pro-inflammatory cytokines triggered by pancreatic injury, where the large aggregation and excessive activation of inflammatory cells leads to pancreatic fibrosis, exacerbating disease progression. Among these inflammatory cells, macrophages, as the main innate immune cells, play a pivotal role in the inflammatory progression of CP.^[25]^ Research has confirmed that CCL23 is mainly expressed by macrophages in the lungs, liver, and pancreas,^[23]^ providing a possible explanation for our findings, namely that CCL23 increases the overexpression of macrophages, thereby increasing the CP risk.

This MR study showed that DNER is a risk factor for CP, and the sensitivity analysis confirmed the robustness of our results. DNER, also known as BET or HE60, is a single channel transmembrane protein attached to peripheral cells that promote the maturation of cell morphology and function.^[26]^ Research indicated that DNER is localized in pro-inflammatory macrophages of human and mice, with elevated expression in macrophage-associated inflammatory markers.^[27]^ Notch 1 is the first notch receptor expressed in the pancreatic epithelium of mouse, and is involved in pancreatic embryonic development and pancreatic carcinogenesis.^[28]^ DNER acts as a novel atypical notch ligand binding to notch 1 and activating the Notch signaling pathway during cells contact.^[29]^ The Notch signaling pathway not only regulates cell life during pancreatic embryonic development, but also promotes pro-inflammatory responses of macrophages.^[30]^ Research has shown that the Notch1 pathway remains activated during the progression of CP.^[28]^ These studies have provided theoretical support for our research.

IL-6 is a multifunctional inflammatory factor with potent pro-inflammatory effects. It is released abundantly after infection and tissue damage to regulate the entire pathological process of inflammatory response, thereby maintaining internal and external balance of the body.^[31]^ Research has confirmed that IL-6 activates PSCS through paracrine and autocrine pathways, promoting the progression of pancreatic fibrosis.^[32]^ As is well known, an important histopathological feature of CP is pancreatic fibrosis. The secretion of IL-6 by inflammatory cells in pancreatic tissue activates the paracrine mechanism and PSCS, promoting chronic inflammation and fibrosis, leading to permanent pancreatitis and exacerbating the occurrence and progression of CP.^[8]^ The above research explains our findings that IL-6 is a risk pathogenic factor for CP.

The MR results suggested that CCL19 and MCP-3 were protective factors for CP. CC chemokines, including CCL19 and MCP-3 (also known as CCL7), are micro secretory proteins connecting the innate and the adaptive immune systems and play a crucial role in the immune system through regulating the migration and localization of immune cells towards inflammatory tissues.^[33]^ CCL19 stabilizes the internal environment and exhibits anti-inflammatory effects, especially in response to internal imbalances, infections, or tissue damage.^[34]^ Research has confirmed its involvement in the M2 polarization of macrophages.^[35]^ MCP-3, or CCL7, is a pleiotropic inflammatory chemokine of anti-infective immunity expressed in endothelial cells that promotes the recruitment of macrophages in vitro, thereby facilitating the clearance of viruses from infected tissues and organs.^[36]^ Research has shown that the activation of MCP-3 contributes to the M2 polarization of macrophages,^[37]^ and M2-polarized macrophages in CP may promote tissue repair and suppress inflammation.^[38]^ In addition, a large amount of evidence suggests that the progression and aggravation of pancreatic fibrosis are associated with CC chemokines. These outcomes provide supportive evidence for our findings.

Our MR analysis confirmed that both IFN-gamma and IL-2 act as protective factors for CP. IFN-gamma(IFN-γ), a cytokine produced by T lymphocytes and NK cells, plays a key factor in host defense response against infectious diseases caused by bacteria, fungi, and protozoa.^[39]^ Known as “macrophage-activating factor,” IFN-γ not only acts as an antiviral factor, but also, recruits macrophages to secrete high-level pro-inflammatory cytokines, hence providing strong protection against intracellular pathogens.^[40]^ The polarization of macrophages regulated by T cells, ILCs, and PSCs is activated to drive fibrosis, in which Th2 cells play a crucial role. Tregs, exhibiting immunosuppressive activity, inhibit Th2 cells and ILC2 in CP, preventing excessive pancreatic fibrosis.^[12]^ A large amount of IFN-γ produced by activated T cells can suppresses the proliferation of Th2 cells,^[41]^ hypothesized to protect against CP by inhibiting pancreatic fibrosis. IL-2 facilitates the immunosuppressive activity of Tregs, which prevents excessive pancreatic fibrosis.^[12]^ IL-2 serves as a growth factor for T cells that can modulate the balance between immune stimulation and immune suppression, thereby inducing and stabilizing the homeostasis of Tregs. It plays a dual role in T cell activation by stimulating the proliferation, differentiation, and maintenance of “conventional T cells, as well as expanding the Treg mass with “immunosuppressive activity.”^[42]^

IL-10, a pleiotropic cytokine, serves as a crucial participant in regulating cell growth and differentiation, as well as modulating inflammatory responses. Recognized as a potent inflammatory suppressor, IL-10 primarily mitigates inflammatory response by activating macrophages to hinder the expression of inflammatory cytokines.^[43]^ Activated macrophages are categorized into 2 phenotypes of M1 and M2. M2 macrophages possess anti-inflammatory properties and play a crucial role in the progression of pancreatic fibrosis. Research has confirmed that IL-10 can alleviate the acute pancreatitis and reduce the risk of post-ERCP pancreatitis. During the progression of CP, IL-10 released by the M2a subtype of M2 macrophages supports cell regeneration and inhibits pancreatic fibrosis.^[12]^ These studies corroborate our findings that IL-10 can reduce the CP risk.

The significance of this study for further research of CP are: Our MR analysis identified CCL23, DNER, and IL-6 as strongly correlated pathogenic factors for CP, while CCL19, IFN-gamma, IL-10, IL-2, and MCP-3 exhibited protective effects, and TNFRSF9 had a bidirectional causal relationship with CP. However, the biological and genetic mechanisms underlying are still not fully elucidated, and require further investigation in future research to provide more effective treatment strategies for patients. Although our MR analysis confirmed the causal relationship between specific inflammatory cytokines and CP, randomized controlled trials and large-sample prospective studies are still needed in future research to comprehensively evaluate the effects of intervention on patients, considering factors such as ethnicity, age, and gender. Our MR analysis, being the largest of its kind, revealed the correlation between inflammatory cytokines and CP, shedding light on the physiological and pathological mechanisms of inflammatory cytokines in CP and suggesting potential diagnostic and therapeutic strategies. Further research might focus on the production of biopharmaceuticals and monoclonal antibodies, offering more effective and comprehensive implementation approaches to the prevention and treatment of CP.

Advantages of this study are the follows. The greatest strength of this study lies in the use of the bidirectional 2-sample MR analysis. MR, grounded in Mendelian laws of inheritance, minimizing confounding factors and unaffected by disease status, thus reducing the possibility of reverse causality. Furthermore, sensitivity analyses with multiple methods have increased the robustness of the results, thereby enhancing the reliability of the research findings. We are the first to use the largest-scale GWAS data for bidirectional MR analysis to investigate the causal relationship between 91 inflammatory cytokines and CP, identifying that CCL23, DNER, and IL-6 are risk factors for CP, while CCL19, IFN-gamma, IL-10, IL-2, and MCP-3 are protective factors, and TNFRSF9 has a bidirectional causal relationship with CP. We applied the largest-scale GWAS data for 2-step MR analysis to explore the mediating role of 1400 metabolites in the causal relationships, confirming that these metabolites do not act as intermediaries.

Limitations of this study are also listed. Limited sample source: The GWAS database in this study is limited to European populations, which fails in representing all humanity, leading to potential biases in other ethnic groups. Insufficient analysis on population differences: It mainly distinguishes different populations based on skin color, hair color, and environmental factors, etc, and lacks stratified analysis of parameters such as age and gender. Other limitations: The study fails to comprehensively cover the relevant fields in its exploration of disease etiology and lacks assessments of drug efficacy.

## 5. Conclusions

The MR analysis conducted in this study has identified that elevated expression levels of CCL23, DNER, and IL-6 increase the CP risk, while CCL19, IFN-gamma, IL-10, IL-2, and MCP-3 play protective roles, and TNFRSF9 has a bidirectional causal relationship with the disease. In addition, among the 9 inflammatory cytokines presenting causal relationships with CP, and elevated level of 6 (CCL19, CCL23, IFN-gamma, IL-2, IL-6, MCP-3) were detected associated with corresponding metabolites. However, there is insufficient evidence to prove that these metabolites have a mediating effect in the causal relationship between the inflammatory cytokines and CP. This study identified inflammatory cytokines with causal effects on CP, providing strong reference for CP research and treatment.

## Acknowledgments

We thank all the researchers who provided data on inflammatory factors, chronic pancreatitis, and blood metabolites, as well as the English translators of this paper.

## Author contributions

**Conceptualization:** Yuqing Wang.

**Data curation:** Chengning Yang, Yuqing Wang, Zuomei Luo.

**Formal analysis:** Chengning Yang.

**Funding acquisition:** Guangwen Chen.

**Investigation:** Lijian Liu, Liqun Li.

**Methodology:** Chengning Yang, Zuomei Luo.

**Project administration:** Guangwen Chen.

**Resources:** Liqun Li.

**Software:** Yuqing Wang.

**Supervision:** Lijian Liu, Guangwen Chen.

**Validation:** Lijian Liu.

**Writing – original draft:** Chengning Yang, Yuqing Wang, Lijian Liu, Zuomei Luo, Nan Chen, Zhu Liu.

**Writing – review & editing:** Liqun Li, Guangwen Chen.

## Supplementary Material


